# Cellular models of genetic diseases

**DOI:** 10.1515/medgen-2021-2095

**Published:** 2021-12-03

**Authors:** Matthias Rath, Ute Felbor

**Affiliations:** University Medicine Greifswald, Department of Human Genetics, Fleischmannstraße 43, D-17475 Greifswald, Germany

There are thousands of rare diseases whose cumulative global prevalence has recently been estimated to be at least 3.5 to 5.9 % [1]. This means that there are hundreds of millions of individuals with rare diseases worldwide. The majority of these disorders is still incompletely understood and cannot be treated effectively. Thus, it is the vision of The International Rare Diseases Research Consortium (IRDiRC) to “*enable all people living with a rare disease to receive an accurate diagnosis, care, and available therapy within one year of coming to medical attention*” [2]. One ambitious goal, which shall be achieved by increasing public awareness and promoting coordinated research, is the approval of 1000 new therapies by 2027 [2]. Although the development of appropriate disease models is a major challenge and often a significant bottleneck to disease mechanism research and drug screening studies, various emerging technologies offer hope for significant progress in bringing bench to bedside. CRISPR/Cas9 genome editing, induced pluripotent stem cell (iPSC) technology, organoid cultures, microfluidic organ-on-a-chip systems, integration of multi-omics approaches, and several others have already taken cell culture models to the next level. The famous quote attributed to the British statistician George E. P. Box, “*All models are wrong, but some are useful*”, will probably also remain to be applicable to modeling complex human diseases *in vitro* for quite some time. However, there has been significant progress in recent years, part of which is presented in this issue.

Best vitelliform macular dystrophy (BVMD) is a perfect example of a rare monogenic disease. *Karolina Plössl*, *Andrea Milenkovic* and *Bernhard H. F. Weber* illustrate how retinal pigment epithelial (RPE) cells differentiated from patient-derived iPSCs have become a valuable research tool to test experimental treatment options and analyze disease mechanism of this *BEST1*-related retinal dystrophy. The authors also discuss limitations of iPSC technology in modeling more complex age-related macular degeneration. Nevertheless, this issue exemplifies that novel cellular and tissue-engineered models are also important for research on more frequent diseases. *Aria Baniahmad* reviews state-of-the-art cancer research with novel three-dimensional cancer models. Tumor spheroids, organoids, and patient-derived xenografts allow investigations into the complex tumor microenvironment and screens for novel anticancer drugs. With a prevalence of 1:500 to 1:1000, hypertrophic cardiomyopathy (HCM) is the most frequent genetic heart disease. *Gökhan Yigit* and *Bernd Wollnik* highlight the important role of human iPSC-based cellular models in studying the pathophysiology of this clinically and genetically heterogeneous disease. Furthermore, they emphasize the importance of emerging novel three-dimensional cardiac models as a more realistic experimental platform for HCM research.

Advances in iPSC technology have also enabled new experimental strategies for neurogenetic diseases that are difficult to study in animal models. *Cagla Cakmak* and *Hans Zempel* review current approaches to model polymerase gamma (POLG)-related disorders with iPSC-derived neurons. Neuronal differentiation of patient-derived iPSCs or stem cells that had been engineered with CRISPR/Cas genome editing allows studies in a disease-related human cellular and neuronal environment. Furthermore, we provide an overview of our own progress in understanding the fundamental mechanisms that drive formation of cerebral cavernous malformations in patients with pathogenic *CCM1*, *CCM2* or *CCM3* germline variants. Modeling the two-step-inactivation of *CCM* genes in human endothelial cells with CRISPR/Cas9 genome editing, we were able to uncover a survival advantage of mutant endothelial cells. In addition, we also review how different cell culture assays might be used for drug repurposing studies. Finally, building the complex neuromuscular junction in cell culture was hardly possible in the past. *Marlen Charlotte Lauffer* illustrates how organ-on-a-chip technologies now allow fascinating insights into the pathophysiologies of neuromuscular disorders. Studies on these miniaturized platforms that allow multiple readouts under more physiological conditions will hopefully help to identify urgently needed novel therapies for this heterogeneous group of disorders.

In summary, this issue gives an overview of recent advances in modeling human diseases in cell culture and highlights upcoming challenges. We thank all authors for their outstanding contributions and hope to increase the visibility of their work and research networking.

## References

[1] Nguengang Wakap S, Lambert DM, Olry A, Rodwell C, Gueydan C, Lanneau V, Murphy D, Le Cam Y, Rath A (2020). Estimating cumulative point prevalence of rare diseases: analysis of the Orphanet database. Eur J Hum Genet.

[2] Austin CP, Cutillo CM, Lau LPL, Jonker AH, Rath A, Julkowska D, Thomson D, Terry SF, de Montleau B, Ardigo D, Hivert V, Boycott KM, Baynam G, Kaufmann P, Taruscio D, Lochmuller H, Suematsu M, Incerti C, Draghia-Akli R, Norstedt I, Wang L, Dawkins HJS, International Rare Diseases Research C (2018). Future of Rare Diseases Research 2017-2027: An IRDiRC Perspective. Clin Transl Sci.

